# Reviewed article: Bastos MES, Nascimento AJF, Soares MTF, Spinelli MBAS, Low ST. Knowledge, attitudes and practices of pregnant women for preventing risks and accidents with their newborns: cross-sectional study, Recife, 2024. Epidemiol Serv Saude. 2026:35;e20240822

**DOI:** 10.1590/S2237-96222026v35e20240822.c

**Published:** 2025-12-08

**Authors:** Mariana Del Grossi Moura

**Affiliations:** 1 Universidade de Sorocaba, Sorocaba, SP, Brazil

## First round comments

Prezados autores,

Agradecemos a submissão do manuscrito RESS-2024-0822 intitulado “Conhecimento, Atitude e Prática da prevenção de riscos e acidentes com o recém-nascido entre gestantes em Recife, Pernambuco, 2024: um estudo transversal” à Revista Epidemiologia e Serviços de Saúde: revista do SUS (RESS).

Após a revisão do seu artigo, identificamos a necessidade de uma revisão substancial para que o manuscrito esteja de acordo com os padrões editoriais da revista e para viabilizar sua publicação.

Além dos pontos detalhados pelos revisores, há necessidade de redução do número de palavras, adequação da linguagem científica com maior objetividade e clareza, reformulação do resumo e da seção de métodos conforme as diretrizes STROBE, padronização estatística (inclusive nos valores apresentados nas tabelas) e reorganização de trechos da Introdução e Discussão para garantir maior coerência e estrutura textual.

Solicitamos que responda a todos os comentários da revisão por pares e forneça uma resposta detalhada explicando de forma clara e objetiva as alterações realizadas (ou justificando a manutenção do texto original, quando pertinente).

Apenas após essa reformulação, o manuscrito poderá ser reconsiderado para publicação. Além disso, ressaltamos que a nova versão poderá ser submetida a nova rodada de avaliação editorial e científica, e que esta decisão inicial não garante a aceitação do manuscrito.

Recommendation: Major revision

## Second round comments

Prezados autores,

Agradecemos a submissão do manuscrito RESS-2024-0822.R1 intitulado “Conhecimento, Atitude e Prática da prevenção de riscos e acidentes com o recém-nascido entre gestantes em Recife, Pernambuco, 2024: um estudo transversal” à Revista Epidemiologia e Serviços de Saúde: revista do SUS (RESS).

Após a revisão do seu artigo, identificamos a necessidade de uma revisão menor para que o manuscrito esteja de acordo com os padrões editoriais da revista e para viabilizar sua publicação.

- Resumo (objetivo): Sugiro ajustar a redação para o plural, uma vez que o estudo envolveu um grupo de gestantes e avalia múltiplos domínios. “Avaliar os conhecimentos, atitudes e práticas das gestantes sobre a prevenção de riscos e acidentes com o recém-nascido”

- Resumo (resultados): é necessário inserir dados numéricos (n ou %) para todas as afirmações feitas. Não usar expressões vagas como “a maioria” ou “destacando-se”. Exemplo: em vez de “mulheres no terceiro trimestre mostraram maior domínio”, escrever “gestantes no terceiro trimestre apresentaram 40,8% de conhecimento adequado sobre engasgo”.

- Resumo (conclusão): A conclusão deve responder de forma direta ao objetivo proposto, com base apenas nos dados apresentados no próprio resumo. Evite generalizações não fundamentadas no resumo, como “a maioria das gestantes” ou “demonstraram conhecimentos inadequados” sem apoio numérico. 

- Introdução: Após ajustar o objetivo no resumo, reproduza a mesma redação no último parágrafo da introdução, garantindo uniformidade entre as seções.

- Resultados/tabelas: Medidas de associação (Phi e V de Cramer): permanecem com 3 casas decimais em algumas tabelas e devem ser ajustadas para duas casas decimais, conforme a padronização solicitada.

- Discussão: reformular o primeiro parágrafo para interpretar os achados centrais (ex.: possíveis implicações, causas prováveis, relevância prática), sem repetir dados numéricos; consolidar todas as limitações metodológicas neste parágrafo: viés de seleção, autorrelato, ausência de avaliação prática, amostragem não probabilística, local único. Evitar justificar a limitação com frases como “esse foi um ponto positivo”; condensar a discussão, evitando circularidade e aprofundando a análise crítica dos achados. Sempre caracterizar os estudos comparados (tipo de estudo, local, população, ano), conforme solicitado; manter a discussão ancorada nos achados do presente estudo, e evitar parágrafos que apenas revisam a literatura.

- Disponibilidade de dados: O link indicado na política editorial da RESS para a lista de repositórios recomendados está atualmente indisponível. Reforçamos, no entanto, que os dados devem estar depositados em repositório confiável, preferencialmente no SciELO Data, com DOI ativo. Solicitamos que os autores: verifiquem se o link para os dados foi inserido corretamente e se está funcionando; confiram se o conteúdo disponibilizado no link corresponde de fato ao conjunto de dados descrito no manuscrito; incluam a citação do dataset nos métodos e a referência completa do conjunto de dados na seção “Referências”, conforme o modelo: Autor(es). Título [dataset]. Ano. DOI

- Tabelas: ajustar o alinhamento dos dados nas tabelas conforme padrão editorial: dados numéricos (frequências, percentuais, medidas estatísticas, p-valores) devem estar alinhados à direita; texto (nomes de variáveis e categorias) deve estar alinhado à esquerda. Substituir os dois-pontos por ponto após o número da tabela (ex.: Tabela 1. ...);

- Tabelas: Os títulos devem ser autossuficientes para ilustração, sem necessidade de consulta ao texto. As Siglas essenciais para a compreensão da ilustração devem aparecer preferencialmente no título, conforme o exemplo: “Tabela 3. Razões de (RP) e intervalos de confiança de 95% (IC95%) brutos e ajustados para o [desfecho] pelas variáveis do estudo. Localização, ano (n=xx )”. As notas de rodapé devem ser utilizadas para esclarecer os resultados apresentados, identificados por letras minúsculas e sobrescritas do alfabeto, em ordem sequencial. As notas abaixo das figuras devem ser identificadas por “Nota:” e separadas por ponto e vírgula.

- Referência 20 está incompleta, inserir epage.

Solicitamos que responda a todos os comentários da revisão por pares e forneça uma resposta detalhada explicando de forma clara e objetiva as alterações realizadas (ou justificando a manutenção do texto original, quando pertinente).

Apenas após essa reformulação, o manuscrito poderá ser reconsiderado para publicação. Além disso, ressaltamos que a nova versão poderá ser submetida a nova rodada de avaliação editorial e científica, e que esta decisão inicial não garante a aceitação do manuscrito.

Recommendation: Minor revision

